# A Systematic Review of the Effects of Hormone Therapy on Psychological Functioning and Quality of Life in Transgender Individuals

**DOI:** 10.1089/trgh.2015.0008

**Published:** 2016-01-01

**Authors:** Jaclyn M. White Hughto, Sari L. Reisner

**Affiliations:** ^1^ The Fenway Institute, Fenway Health, Boston, Massachusetts.; ^2^ Department of Chronic Disease Epidemiology, Yale School of Public Health, New Haven, Connecticut.; ^3^ Division of General Pediatrics, Boston Children's Hospital/Harvard Medical School, Boston, Massachusetts.; ^4^ Department of Epidemiology, Harvard T.H. Chan School of Public Health, Boston, Massachusetts.

**Keywords:** clinical care, gender dysphoria, gender transition, mental health, transgender

## Abstract

**Objectives:** To review evidence from prospective cohort studies of the relationship between hormone therapy and changes in psychological functioning and quality of life in transgender individuals accessing hormone therapy over time.

**Data Sources:** MEDLINE, PsycINFO, and PubMed were searched for relevant studies from inception to November 2014. Reference lists of included studies were hand searched.

**Results:** Three uncontrolled prospective cohort studies, enrolling 247 transgender adults (180 male-to-female [MTF], 67 female-to-male [FTM]) initiating hormone therapy for the treatment of gender identity disorder (prior diagnostic term for gender dysphoria), were identified. The studies measured exposure to hormone therapy and subsequent changes in mental health (e.g., depression, anxiety) and quality of life outcomes at follow-up. Two studies showed a significant improvement in psychological functioning at 3–6 months and 12 months compared with baseline after initiating hormone therapy. The third study showed improvements in quality of life outcomes 12 months after initiating hormone therapy for FTM and MTF participants; however, only MTF participants showed a *statistically* significant increase in general quality of life after initiating hormone therapy.

**Conclusions:** Hormone therapy interventions to improve the mental health and quality of life in transgender people with gender dysphoria have not been evaluated in controlled trials. Low quality evidence suggests that hormone therapy may lead to improvements in psychological functioning. Prospective controlled trials are needed to investigate the effects of hormone therapy on the mental health of transgender people.

## Introduction

Transgender people have a gender identity or expression that differs from their sex assigned at birth. Research documents high prevalence of depression, anxiety, and suicidal ideation among transgender individuals relative to the general population.^1–3^ Many transgender people experience psychological distress related to the discrepancy between their birth sex and felt a sense of being male, female, or otherwise gender nonconforming.^4^ Some individuals experience gender-related psychological distress at such an extreme level that their distress meets criteria for a formal psychiatric diagnosis—*Gender Dysphoria*.^4^ The critical element of gender dysphoria is the presence of clinically significant distress associated with the strong and persistent difference between one's expressed/experienced gender and one's sex assigned at birth.^5^ The World Professional Association of Transgender Health's Standards of Care for the Health of Transsexual, Transgender, and Gender Nonconforming People (Version 7) lists social transition (living full or part-time in one's gender identity), psychotherapy, surgery, and hormone therapy as treatment options for individuals with gender dysphoria.^6^ While each person's treatment plan is individualized, and may include one or multiple interventions, hormone therapy is generally the first medical intervention accessed by individuals with gender dysphoria who seek to masculinize or feminize their body to be consistent with their gender identity.^6^

Feminizing or masculinizing hormone therapy is the administration of exogenous endocrine agents to induce changes in physical appearance.^6^ Since hormone therapy is inexpensive relative to surgery and highly effective in the development of secondary sex characteristics (e.g., facial and body hair in female-to-male [FTM] individuals or breast tissue in male-to-females [MTFs]), hormone therapy is often the first, and sometimes only, medical gender affirmation intervention accessed by transgender individuals looking to develop masculine or feminine characteristics consistent with their gender identity. In some cases, hormone therapy may be required before surgical interventions can be conducted.^6^

Changing one's physical characteristics through hormone therapy is considered medically necessary for many transgender individuals and may relieve the psychological distress associated with gender dysphoria, reduce psychiatric comorbidities, and improve patients' quality of life.^6^ The efficacy of hormone therapy in relieving psychiatric distress related to gender dysphoria has largely been inferred through clinical practice and low quality evidence.^7^ In 2008, the first systematic review exploring the relationship between hormone therapy and the mental health of transgender individuals was conducted.^7^ Published in 2010, the review found that hormone therapy in individuals with gender identity disorder (the DSM-IV diagnostic name for gender dysphoria) *likely* improves gender dysphoria, psychological function, comorbidities (e.g., depression, anxiety, and suicidality), sexual functioning, and overall quality of life.^7^ However, the quality of the empirical evidence was very low. All of the reviewed studies pertaining to psychological functioning were nonrandomized and largely cross-sectional designs. Moreover, the majority of the studies evaluated hormone therapy in concert with sex reassignment surgery and so an evaluation of the specific relationship between hormone therapy and the psychological functioning of transgender individuals independent of surgical interventions was not possible. Since 2008, several higher quality studies exploring the relationship between hormone therapy and psychological functioning in transgender individuals have been published, thus an updated systematic evaluation of the research literature is warranted.

The primary objectives of this review were to locate and describe studies examining the relationship between hormone therapy and the psychological functioning of transgender individuals; determine the risk of experiencing psychiatric distress in transgender individuals receiving hormone therapy compared with those not receiving hormone therapy or the general population; examine whether transgender individuals with poor mental health experience an improvement in their mental health status after the initiation of a hormone regimen; and assess whether any differences in the strength and direction of the association between hormone therapy and psychological functioning exist by gender identity (i.e., for MTF vs. FTM subjects). Additionally, the review aimed to assess the strength of evidence showing a relationship between hormone therapy and healthy psychological functioning in transgender individuals and identify future research and clinical practice needs.

## Methods

PRISMA^8^ reporting guidelines were used in the development of this protocol-driven report. The protocol was not published, but is available upon request.

### Eligibility criteria

Studies were eligible for this review if they enrolled transgender or transsexual (or another term used to describe individuals with a gender identity different from their assigned sex at birth) individuals who were seeking hormone therapy to masculinize or feminize the body. All participants had been diagnosed with having gender identity disorder (prior diagnostic name for gender dysphoria).

Because the goal was to assess changes in psychological functioning and quality of life after initiating hormone therapy, only studies with a longitudinal design were included in the review. No randomized controlled trials were identified. All included studies were nonrandomized (observational) and uncontrolled, prospective cohort studies. One-time cross-sectional studies, single case reports, case series, review articles, commentaries, letters, and studies that did not contain original data were excluded. Studies were included regardless of sample size. Only English language publications were included in the search. Studies following participants for less than 3 months were excluded.

Interventions were limited to the administration of feminizing or masculinizing hormone therapy. Due to the wide variation of doses and types of hormones, all forms of hormone administration (e.g., self or provider injection, oral pill, intramuscular, transdermal patch or gel, or subcutaneous implant), dosing levels, dose frequency, and types (e.g., estrogen, androgen-reducing medications, progestins, testosterone, bioidentical, and compounded hormones) were included. Psychiatric disorders (DSM diagnosis or clinically significant symptomology), general psychological functioning, and general quality of life outcomes were assessed.

### Study identification

The first author consulted an expert reference librarian to design and conduct the electronic search strategy with input from the second author and collaborators with expertise in conducting systematic reviews. To identify eligible studies, electronic databases (Ovid MEDLINE, Ovid PsycInfo, and PubMed) were searched from inception to November 2014. Controlled vocabulary supplemented with keywords was used to define the concept areas (transgender/transsexual, hormone therapy, psychiatric disorder, psychological functioning, and quality of life). A hand search of the bibliographies of retrieved articles was also conducted. A detailed list of subject headings and text words is available upon request.

The first author examined abstracts and titles from the initial search to identify studies that appeared to meet the inclusion criteria. Abstracts were screened twice for inclusion. The full article was then obtained for all studies appearing to meet inclusion criteria or in instances where there was insufficient information from the title, keywords, and abstract to make a clear decision. The studies meeting the inclusion criteria were checked for validity assessment (see the Quality assessment section) and data extraction.

### Data collection

Data were extracted for the studies that met inclusion criteria, and then entered into RevMan5. Data were extracted using specially developed data extraction forms to ensure standardization, which included the following headings:

#### Participant characteristics

Age, birth sex, gender identity, income, education, and psychiatric comorbidities (all at baseline).

#### Study characteristics

Recruitment method, inclusion/exclusion criteria, setting, allocation procedure, sample size analyzed, study design features, and follow-up period.

#### Intervention

Description of intervention, mode and frequency of delivery, number, and explanation for any dropouts.

#### Outcomes

Description of measures used, continuous/dichotomous nature, and validated or unvalidated method of administration.

#### Statistical results

Adjusted and unadjusted effect estimates (if available) and measures of variability, frequency counts for dichotomous variables, and number of patients.

#### Confounding

Specific confounders included the following factors, which could impact the mental health of participants independently of hormone therapy: receiving psychotherapy in addition to hormone therapy; accessing other medical gender affirmation technologies (i.e., silicon injections, surgeries, electrolysis) in addition to hormone therapy; possessing any underlying treated or untreated comorbid psychiatric conditions; relationship factors and social support (positive or negative relationships with family, friends, sexual partners); discrimination experiences (in employment, housing, etc.); barriers and facilitators to healthcare access (insurance coverage for hormones or surgeries; feasibility in accessing trained and competent providers, etc.); prior or concurrent use of street-based hormones; and patient characteristics (age, income, level of education).

### Quality assessment (risk of bias)

In consultation with an expert in systematic reviews, the first author assessed the risk of bias for each study and reported the bias in the Risk of Bias table according to the Cochrane Handbook for Systematic Reviews of Interventions.^9^ Specifically, sequence generation, allocation sequence concealment, blinding, incomplete outcome data, selective outcome reporting, and other potential sources of bias were assessed. Items were assessed by first providing a description of what occurred in the study and then providing a judgment on the adequacy of the study as it relates to each item. When assessing bias, studies were scored as low risk of bias, high risk of bias, and unclear for studies with unclear or unknown risk of bias. Since this tool was developed specifically for RCTs, items from the Downs and Black instrument^10^ and the Newcastle-Ottawa Scale^11^ were included in the assessment. The authors intended to explore selective outcome reporting through sensitivity analyses in cases where missing data were likely to introduce serious bias; however, none of these factors were reported in the included studies.

### Measures of treatment effect

For prospective cohort studies, statistically significant (*p*<0.05) within-group pre–post changes (%) were analyzed. The summary measure was the weighted mean difference (standardized mean difference [SMD]) in change in mental health from baseline to follow-up among transgender individuals initiating hormone therapy. For outcomes based on a single study, mean change scores were reported.

The Cochrane Collaboration statistical guidelines were followed for data synthesis.^9^ Data were collated into evidence tables and grouped according to each outcome. The reported primary and secondary outcome variables were grouped as follows: Primary: psychological functioning, depression, anxiety, somatization, interpersonal sensitivity, hostility, phobic anxiety; and Secondary: quality of life.

### Data analysis

Data were analyzed using RevMan software and reported according to Cochrane Collaboration criteria.^9^ A summary statistic for each study was created where possible. A generic inverse variance method was used to create a weighted average for each study according to the amount of information in each study and a fixed-effects model was employed. For continuous data, pooled outcomes were expressed as SMDs with associated 95% confidence intervals (CIs) when outcomes used different scales or different versions of the same scale. When outcomes used the same scale, the weighted mean difference was used and 95% CIs reported.

To avoid a unit of analysis error, several different outcomes based on different periods of follow-up were defined and not combined in a meta-analysis. When missing data were found, the data were assumed to be missing at random. Only available data were analyzed and missing data were ignored.

Inconsistency in treatment effects across studies was quantified using the I^2^ statistic, which represents the proportion of variability across trials that is not attributable to random error or chance.^9^ Assessment of heterogeneity was made based on the following levels: none <25%, low=25–49%, moderate=50–74%, and high=75%+.^8^ Since study outcomes and measurement were too heterogeneous, a narrative synthesis was performed.

## Results

**FIG. 1.**
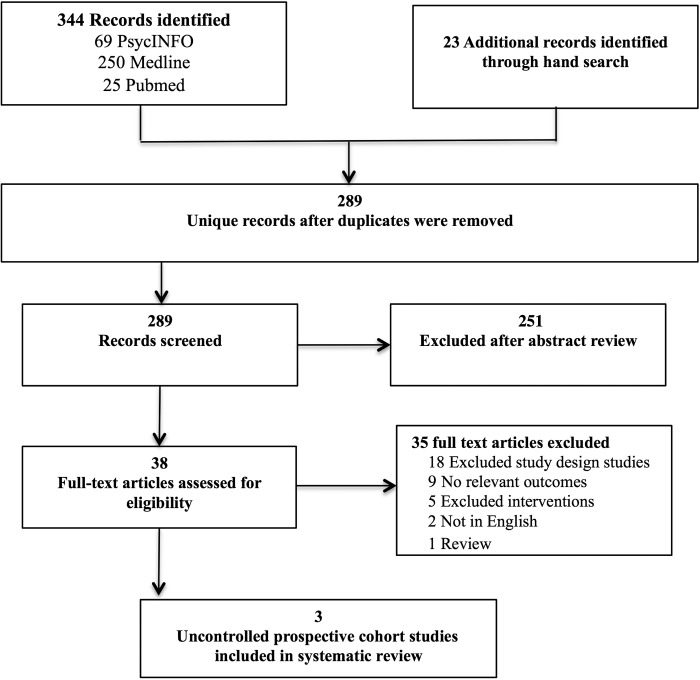
Flow diagram of study inclusion for systematic review of masculinizing and feminizing hormone therapy and psychological functioning and quality of life in transgender patients.

Figure 1 depicts the study selection process. Three studies that met inclusion criteria were identified.^12–14^ Of the three studies, data were available for a total of 154 participants (two studies) for the primary psychological functioning outcomes;^12,14^ one study reported on secondary quality of life outcomes (*n*=83).^13^ Lack of data and variation in follow-up period of assessment time points precluded meta-analysis for the reviewed studies.

### Study characteristics

In all three studies, a cohort of transgender patients initiating hormone therapy was followed longitudinally after initiating hormone therapy at baseline. The timing of follow-up for these studies ranged from 3 to 6 months and 12 months after baseline. One study used a 3–6-month follow-up^14^ and two used a 12-month follow-up.^12,13^ One study^14^ followed participants beyond 12 months after completion of gender affirmation surgery; however, this time point was not included in the analyses as per the protocol. All studies lacked a control group.

The three studies enrolled 247 participants (180 MTF, 67 FTM). Only two studies reported the age of participants,^12,13^ and the mean age for MTF individuals across studies was similar to FTM individuals (mean age 31 and 30, respectively). All three of the included studies originated in Europe (two in Italy; one in Belgium) and were published in English.

Participants were all recruited from specialty gender identity clinics serving patients diagnosed with having gender identity disorder (prior DSM diagnosis for gender dysphoria). Patients were recruited into the study during a routine visit and all were seeking surgical gender affirmation surgery (i.e., sex reassignment surgery).

All three studies administered hormone therapy following diagnosis of gender identity disorder. All participants lived full-time in their gender role (socially transitioned) and were receiving concurrent psychotherapy. Specific details of the hormone regimen were reported by two studies, which used similar regimens. Both studies administered either transdermal or oral estrogen and antiandrogens to MTF patients, although dosing varied.^12,13^ For FTM patients, both studies used intramuscular injections of testosterone, although dosing quantity and frequency differed and one study provided daily low-dose transdermal gel testosterone at the beginning of the treatment period (10–15 days).^13^

The outcomes were ascertained by a self-reported questionnaire in all three studies. Two studies administered assessments in the clinic and one mailed the questionnaire to the participants at follow-up, resulting in missing data. Two studies used the Symptom Checklist-90 (SCL-90) to assess psychological functioning.^12,14^ However, one study^12^ used the Italian revised version (SCL-90-R)^15^ and the other study^14^ used the traditional Dutch-adapted version.^16^ Two studies assessed anxiety;^12,14^ however, only one^12^ used a validated scale (Zung Self-Reported Anxiety Scale).^17^ One study^12^ also measured depression using a second validated depression measure (Zung Depression Scale).^18^ Only one study assessed quality of life as a secondary outcome^13^ and used the World Health Organization Quality of Life-100 (WHOQOL-100) questionnaire.^19^

### Outcomes

Table 1 describes the summary of findings for reported outcomes in included studies.

**Table 1.** **Hormone Therapy at Follow-Up Compared to No Hormone Therapy at Baseline for Improving Psychological Functioning and Quality of Life in Transgender Patients**

Outcomes	Relative effect (95% CI)	No. of participants (studies)	Quality of the evidence	Comments
Psychological functioning: global psychiatric distress (SCL-90)				
SCL-90-Dutch^14^				
Overall psychoneurotic distress standardized mean difference 3–6 months	SMD=−0.88 (−1.29, −0.48)	154 (2 studies)	Low ⊕⊕⊕⊖	Two uncontrolled prospective cohort studies investigated hormone therapy and found that global psychiatric distress scores were significantly lower after treatment.
SCL-90-R Italian^12^				
GSI Standardized mean difference 12 months	SMD=−0.52 (−0.79, −0.25)			
Psychological functioning: depression (SCL-90)				
SCL-90-Dutch^14^				
Standardized mean difference 3–6 months	SMD=−0.89 (−1.30, −0.48)	154 (2 studies)	Low ⊕⊕⊕⊖	Two uncontrolled prospective cohort studies investigated hormone therapy and found that depression scores were significantly lower after treatment.
SCL-90-R Italian^12^				
Standardized mean difference 12 months	SMD=−0.51 (−0.78, −0.24)			
Psychological functioning: anxiety (SCL-90)				
SCL-90-Dutch^14^				
Standardized mean difference 3–6 months	SMD=−0.78 (−1.18, −0.38)	154 (2 studies)	Low ⊕⊕⊕⊖	Two uncontrolled prospective cohort studies investigated hormone therapy and found that anxiety scores were significantly lower after treatment.
SCL-90-R Italian^12^				
Standardized mean difference 12 months	SMD=−0.66 (−0.93, −0.38)			
Psychological functioning: somatization (SCL-90)				
SCL-90-Dutch^14^				
Standardized mean difference 3–6 months	SMD=−0.64 (−1.04, −0.24)	154 (2 studies)	Low ⊕⊕⊕⊖	Two uncontrolled prospective cohort studies investigated hormone therapy and found that somatization scores were significantly lower after treatment.
SCL-90-R Italian^12^				
Standardized mean difference 12 months	SMD=−0.38 (−0.65, −0.10)			
Psychological functioning: interpersonal sensitivity (SCL-90)				
SCL-90-Dutch^14^				
Standardized mean difference 3–6 months	SMD=−0.70 (−1.10, −0.30)	154 (2 studies)	Low ⊕⊕⊕⊖	Two uncontrolled prospective cohort studies investigated hormone therapy and found that interpersonal sensitivity scores were significantly lower after treatment.
SCL-90-R Italian^12^				
Standardized mean difference 12 months	SMD=−0.47 (−0.75, −0.20)			
Psychological functioning: hostility (SCL-90)				
SCL-90-Dutch^14^				
Standardized mean difference 3–6 months	SMD=−0.31 (−0.70, 0.08)	154 (2 studies)	Low ⊕⊕⊕⊖	Two uncontrolled prospective cohort studies investigated hormone therapy and found that hostility scores were lower after treatment, although only one reached statistical significance.
SCL-90-R Italian^12^				
Standardized mean difference 12 months	SMD=−0.34 (−0.61, −0.07)			
Psychological functioning: phobic anxiety (Agoraphobia) (SCL-90)				
SCL-90-Dutch^14^				
Standardized mean difference 3–6 months	SMD=−0.42 (−0.81, −0.03)	154 (2 studies)	Low ⊕⊕⊕⊖	Two uncontrolled prospective cohort studies investigated hormone therapy and found that phobic anxiety scores were significantly lower after treatment.
SCL-90-R Italian^12^				
Standardized mean difference 12 months	SMD=−0.37 (−0.64, −0.10)			
Psychological functioning: depression (Zung Scale)				
Zung depression^12^				
Mean difference 12-month follow-up	−8.06 (−10.91, −5.21)	107 (1 study)	Moderate ⊕⊕⊕⊖	One study assessed changes in mean anxiety scores and found significant reductions in anxiety at 12-month follow-up, with scores now in the normal range.
Psychological functioning: anxiety (Zung Scale)				
Zung anxiety^12^				
Mean difference 12-month follow-up	−7.01 (−9.50, −4.52)	107 (1 study)	Moderate ⊕⊕⊕⊖	One study assessed changes in mean depression scores and found significant reductions in depression at 12-month follow-up, with scores now in the normal range.
Quality of Life (WHOQOL-100)				
WHOQOL-100^13^				
Mean difference 12-month follow-up full sample, MTF, FTM	8.3, 9.7, 5.5 (N/A)	107 (1 study)	Very low ⊕⊖⊖⊖	One study assessed mean changes in quality of life by subgroup; however, lack of reported standard deviation and *p*-values meant that CIs for full sample could not be calculated. Only MTF participants had a statistically significant increase (*p*<0.05) in overall quality of life at 12-month follow-up.

Patient or population, transgender individuals with Gender Dysphoria; settings, European gender identity clinic; intervention, hormone therapy; comparison, no hormone therapy at baseline. Due to heterogeneity in the length of follow-up for all outcomes, the overall effect estimates are not presented. Formal GRADE^20^ evaluation was not conducted; however, quality was ranked using GRADE terminology: high quality, further research is very unlikely to change our confidence in the estimate of effect; moderate quality, further research is likely to have an important impact on our confidence in the estimate of effect and may change the estimate; low quality, further research is very likely to have an important impact on our confidence in the estimate of effect and is likely to change the estimate; very low quality, we are very uncertain about the estimate.

CI, confidence interval; FTM, female-to-male; GSI, Global Severity Index; MTF, male-to-female; SCL-90, Symptom Checklist-90; SMD, standardized mean difference; WHOQOL-100, World Health Organization Quality of Life-100.

#### Psychological functioning

Two studies (*n*=154) assessed psychological functioning and both used continuous outcomes as measured by the SCL-90.^12,14^ Two studies used different versions of the SCL-90 (revised version, Italian version;^12^ Dutch-adapted SCL-90^14^), with one study assessing outcomes at 3–6 months posthormone initiation^14^ and the other assessing outcomes at 12 months posthormone initiation.^12^ One study^12^ used additional measures for anxiety and depression (Zung scale), the outcomes of which are reported below. All psychological functioning outcomes were measured continuously with mean change scores reported for the SCL-90 and for the Zung depression and anxiety scales (see summary of findings: Table 1).

#### Global score of psychological functioning

The SCL-90-R Italian version provides a global score of psychological functioning (Global Severity Index), while the SCL-90 Dutch version provides an overall psychoneurotic distress score, which are similar, but scored differently. Both studies measuring overall psychiatric distress saw a significant change in global distress scores at follow-up: SMD=−0.52 [95% CI: −0.79, −0.25] (12 months of follow-up)^12^ and −0.88 [95% CI: −1.29, −0.48] (3–6 months of follow-up).^14^

#### Depression

Participants in both studies had depression scores in the normal range at baseline as indicated by the SCL-90-R Italian version depression subscale, Zung Depression Scale,^12^ and SCL-90 Dutch depression subscale.^14^ Nonetheless, the SCL-90-R Italian version^12^ and SCL-90 Dutch version each showed statistically significant reductions in depression posthormone therapy across the two studies with an SMD of −0.51 [95% CI: −0.78, −0.24] (12-month follow-up)^12^ and −0.89 [95% CI: −1.30, −0.48] (3–6 months of follow-up).^14^ One study also assessed depression scores using the Zung Depression Scale and saw a similar statistically significant reduction at 12-month follow-up (mean difference=−8.06 (95% CI: −10.91, −5.21).^12^ Meta-analysis of these two studies was precluded due to statistical heterogeneity (I^2^=0%) and disparate follow-up periods.

#### Anxiety

Two studies measured changes in anxiety scores from baseline to 3–6-month^14^ and 12-month^12^ posthormone therapy initiation using the SCL-90 anxiety subscale (SCL-90-R Italian version;^12^ SCL-90 Dutch version^14^). One study also used an additional validated continuous measure of anxiety (Zung Anxiety Scale).^12^

The SCL-90 anxiety subscales showed higher than normal anxiety for participants in both studies at baseline (mean=1.05, SD=0.94; normal <1.0;^12^ mean=17.0, SD=6.4; general population mean=12.8, SD=4.4).^14^ At follow-up, both studies saw a statistically significant reduction in anxiety with scores in the normal range at 3–6-month follow-up (mean=12.4, SD=5.1)^14^ and 12-month follow-up (mean=0.54, SD=0.56),^12^ as well as significant standardized mean change scores (−0.66 [95% CI: −0.93, −0.38];^12^ −0.78 [95% CI: −1.18, −0.38]^14^).

One study also showed a reduction in anxiety using a different continuous measure (Zung Anxiety) from above the normal range at baseline (mean=44.91, SD=9.59; normal=24–44) to within the normal range at 12-month follow-up (mean=37.90, SD=8.97)^12^ and this change was highly significant (mean difference=−7.01 [95% CI: −9.50, −4.52]).

#### Somatization

Two studies assess somatization as a subscale measure in the SCL-90 (SCL-90-R Italian version;^12^ SCL-90 Dutch version^14^). One study had somatization scores within the normal range at baseline (mean=0.54, SD=0.59; normal <1.0).^12^ The other study had significantly higher baseline somatization scores compared with the general population at baseline (mean=18.6, SD=6.7; general population mean=16.7, SD=5.3);^14^ however, scores remained significantly higher than the general population even at 3–6-month follow-up (mean=15.2, SD=2.7). Nonetheless, both studies saw a significant reduction in somatization scores posthormone therapy initiation as measured by mean change scores at 3–6-month follow-up (−0.64 [95% CI: −1.04, −0.24])^14^ and 12-month follow-up (−0.38 [95% CI: −0.65, −0.10]).^12^

#### Interpersonal sensitivity

Two studies assessed interpersonal sensitivity using the SCL-90 (SCL-90-R Italian version;^12^ SCL-90 Dutch version^14^). Participants in one study had significantly higher scores than the general population at baseline (mean=31.8, SD=11.7; general population mean=24.1, SD=7.6).^14^ The other study had normal interpersonal sensitivity scores at baseline (mean=0.97, SD=0.82; normal <1.0).^12^ Participants in both studies evinced a significant reduction in standardized mean change scores posthormone initiation at 3–6-month follow-up (SMD=−0.70 [95% CI: −1.10, −0.30])^14^ and 12-month follow-up (SMD=−0.47 [95% CI: −0.75, −0.20]).^12^ One study showed a reduction in interpersonal sensitivity scores that no longer differed significantly from those of the general population at 3–6-month follow-up (mean=16.6, SD=7.0; general population mean=24.6, SD=7.9).^14^ While both studies^12,14^ lacked statistical heterogeneity (I^2^=0%), the interpersonal sensitivity outcomes were measured at different time points (12 months;^12^ 3–6 months^14^), making meta-analysis inappropriate.

#### Hostility

Using the SCL-90, two studies assessed hostility (SCL-90-R Italian version;^12^ SCL-90 Dutch version).^14^ Participants in both studies had hostility scores in the normal range, consistent with that of the general population (mean=0.57, SD=0.69; normal <1.0;^12^ mean=8.2, SD=3.0; general population mean=7.2, SD=2.1).^14^ Nonetheless, both studies showed a reduction in hostility scores posthormone therapy initiation, although the reduction was not statistically significant at 3–6 month follow-up (SMD=−0.31 [95% CI: −0.70, −0.08])^14^ and the other was statistically significant at 12-month follow-up (SMD=−0.34 [95% CI: −0.61, −0.07]).^12^ One study demonstrated a reduction in scores to a similar level as the general population at 3–6 month follow-up (mean=7.4, SD=2.0; general population mean=7.2, SD=2.1).^14^

#### Phobic anxiety

Two studies assessed phobic anxiety using the SCL-90 (SCL-90-R Italian version;^12^ SCL-90 Dutch version).^14^ Participants in one study had phobic anxiety scores in the normal range before initiating hormone therapy (mean=0.53, SD=0.61; normal <1.0).^12^ The other study had phobic anxiety called agoraphobia in the SCL-90 Dutch version^14^ scores that were significantly higher than the general population at baseline (mean=9.5, SD=4.2; general population mean=7.9, SD=2.3).^14^

Both studies saw a statistically significant change in phobic anxiety scores from baseline to follow-up (SMD=−0.37 [95% CI: −0.64, −0.10];^12^ SMD=−0.42 [95% CI: −0.81, −0.03]),^14^ with one study showing a reduction in phobic anxiety in scores to a level that was similar to those of the general population (mean=8.1, SD=1.8; general population mean=7.9, SD=2.3).^14^

#### Quality of life

Only one study (*n*=83) assessed quality of life using the validated WHOQOL-100 questionnaire. Mean baseline scores and 12-month postintervention follow-up scores are reported overall and by subgroup (MTF/FTM). The study reported data for subgroups (MTF/FTM) and the mean follow-up scores were calculated for the full sample. The mean standard deviations for both subgroups were not reported and the *p*-value from the paired *t*-tests was only reported for the MTF subgroup, thus it was not possible to calculate a standard deviation for the full sample and compare pre/post means. Thus, findings must be considered in light of lack of reported data. See summary of findings (Table 1).

One study reported on general quality of life.^13^ At baseline, general quality of life, as measured through the WHOQOL-100, was in the normal/good range (i.e., ≥50) at 62.74. At follow-up, the quality of life score increased to 71.08 (max=100); however, the significance of this increase cannot be determined. Among MTF participants, paired t-tests showed a significant increase in overall quality of life from 62.5 to 72.2 (*p*<0.05) at 12-month follow-up. For FTM participants, increases were observed (63.25 [baseline]; 68.75 [follow-up]); however, the increase in overall quality of life was not significant.^13^ Given that only one study reported on quality of life outcomes, meta-analysis was not possible.

### Methodological quality

**FIG. 2.**
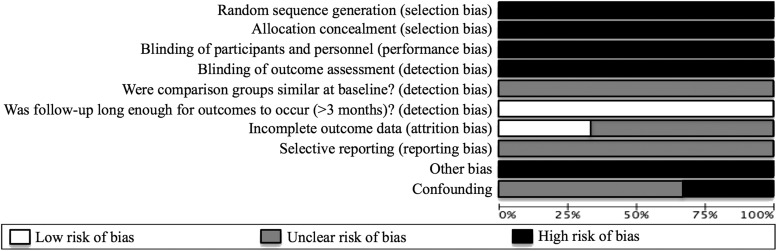
Risk of bias: Assessment of each risk of bias item presented as percentages across all included studies.

See summary of findings (Table 1) and risk of bias (Fig. 2). The overall quality of evidence is low. While the studies used a prospective design, which is better quality than one-time cross-sectional studies, all studies lacked a control group. No studies used a randomized design. Moreover, all outcomes were based on self-report, which is subject to bias. However, all the measures used were based on psychometrically valid and reliable scales.

One confounding factor is that all studies provided psychotherapy to patients as part of standard of care for their clinic and all patients socially transitioned while accessing hormone therapy. While concurrent psychotherapy is often accessed by transgender patients on hormone therapy, and transgender patients have generally socially transitioned before initiating hormones, the effects of psychotherapy and social transition on mental health outcomes could have masked the true effects of hormone therapy on the mental health of patients. The studies were careful, however, to exclude participants with serious untreated psychiatric comorbidities (e.g., schizophrenia, substance abuse, and personality disorder).

Many studies failed to assess important confounders such as access to other medical gender affirmation technologies (e.g., surgeries) and none of the studies adjusted for confounders in their results such as the use of psychotropic medications.

## Discussion

Although the studies differ with regard to outcome measures and most studies have methodological shortcomings, the three prospective cohort studies reviewed here offer provisional conclusions about the effects of hormone therapy on the psychological functioning of transgender individuals accessing it. Two studies^12,14^ reported on psychological functioning and found a statistically significant reduction in depression, somatization, interpersonal sensitivity, anxiety, hostility, and phobic anxiety/agoraphobia after initiating hormone therapy, with one study observing significant results 3–6 months posthormone initiation and the other 12 months posthormone initiation. There is insufficient evidence about whether changes in quality of life occur for FTM individuals who initiate hormone therapy. However, low quality evidence suggests that quality of life may be improved for MTF individuals accessing hormone therapy.

Findings support and extend the findings of another review,^7^ which provided very low quality evidence that hormone therapy may improve the mental health of transgender people. However, the prior systematic review, which included 28 studies, assessed the effects of hormone therapy together *with* sex reassignment surgery on mental health, psychological functioning, sexual functioning, and quality of life and thus was unable to parse out the effects of hormone therapy separately from surgical interventions.

Given that many transgender people may never access sex reassignment surgery, it was important to study the effects of hormones alone with regard to mental health and quality of life outcomes. Moreover, the majority of studies included in the prior review were cross-sectional and none of the studies assessing mental health or psychological functioning used prospective study designs with a follow-up period of 3 months or more. Furthermore, the only studies that included a control group in the prior review assessed sex reassignment surgery and hormone therapy together, without comparing hormone therapy with an untreated control. Nonetheless, the prior review did find that the studies assessing sex reassignment surgery together with hormone therapy were strongly associated with improved psychological functioning,^7^ thus findings from the current review extend these results.

Like the previous systematic review,^7^ results of this review demonstrate low quality evidence (previous review demonstrated *very* low quality evidence), suggesting that hormonal therapies given to individuals diagnosed with having gender identity disorder (i.e., gender dysphoria) improve psychological functioning. However, unlike the previous review, this review is unable to offer conclusive evidence regarding the effects of hormone therapy on quality of life for transgender individuals overall.

Contrary to the prior review,^7^ which found that MTF transgender people may have worse outcomes than FTM individuals, the study reviewed here found that MTF individuals had statistically significant improvements in quality of life 12 months after initiating hormone therapy, while FTM individuals did not experience increases that reached statistical significance, which was likely due to the small sample size.^13^ Additional research is needed to confirm quality of life results overall and by gender subgroups.

Limitations regarding potential sources of bias in the review process should be noted. Three databases were used to identify studies for inclusion in this review and the search was limited to studies published in English. These search limitations may have resulted in the loss of relevant studies and influenced the results of the review. However, the electronic search was supplemented by checking references and citations of other articles, which yielded additional studies. Moreover, while the field of transgender health is truly global, English represents the predominant scientific publication language, thus significant findings, using less biased observational results, would have been likely to be published in English.

Nonstatistically significant findings may not have been published in English (i.e., publication bias). Nonetheless, it is feasible that all relevant studies have been identified. Additionally, study identification was completed by a single reviewer (the first author) in consultation with an expert reference librarian and researchers with expertise in systematic reviews. While the reviewer thoroughly conducted the search and review process, it is possible that studies that met inclusion criteria were not included in the review or were subject to a single reviewer's biases.

The restriction to prospective cohort studies also meant that the reviewer was unable to consider data from other types of evaluations of hormone therapy interventions such as one-time cross-sectional studies or qualitative evaluations. Although prospective cohorts are a stronger design than cross-sectional studies due to temporal ordering of exposure and outcomes, the cohort studies in this review were subject to a number of biases and other limitations (see the risk of bias section above and Fig. 2).

Uncontrolled prospective cohort studies suggest that hormonal therapies given to individuals diagnosed with having gender identity disorder (i.e., gender dysphoria) likely improve psychological functioning 3–12 months after initiating hormone therapy. Findings from the review support current clinical care guidelines such as the WPATH Standards of Care,^6^ which recommend the use of hormone therapy as a treatment option to reduce gender dysphoria. Future research should assess the effects of hormone therapy on the mental health of transgender individuals using more robust study designs, including those which utilize clinician-delivered mental health outcome measures, longitudinal designs with control groups, and those examining U.S.-based transgender people over time.

## Abbreviations Used

CI: confidence interval
FTM: female-to-male
MTF: male-to-female
SCL-90: Symptom Checklist-90
SMD: standardized mean difference
WHOQOL-100: World Health Organization Quality of Life-100
